# Structural characterization of stripe rust progress in wheat crops sown at different planting dates

**DOI:** 10.1016/j.heliyon.2020.e05328

**Published:** 2020-11-13

**Authors:** Bita Naseri, Homayoon Kazemi

**Affiliations:** a Plant Protection Research Department, Kermanshah Agricultural & Natural Resources Research & Education Center, Iran; b Iranian Research Institute of Plant Protection, Iran

**Keywords:** Cereals, Epidemiology, Multivariate analysis, Yellow rust, Agricultural technology, Agronomy, Systems biology, Microbiology

## Abstract

An advanced insight into characterizing stripe rust progress curves is required to improve accuracy and efficiency of future research for disease measurement and estimation purposes. The rate of stripe rust increase in wheat crops is highly variable, resulting in variations and uncertainties in evaluating disease progress over time. This variability was described by fitting standard curves to disease severity data collected over a four-season experiment to identify effective disease curve elements in Iranian wheat cultivars planted at different dates. Gaussian curves appeared to be the best fitted models for all four growing seasons. Three Gaussian parameters in combination with the area under the disease progress curve (AUDPC), disease onset time, maximum disease incidence and severity were then considered to describe the rate of disease increase. Based on *H*-tests of Kruskal-Wallis one-way analysis of variance, cultivar and planting date significantly affected AUDPC, maximum disease incidence and severity. There were significant correlations between continuous disease descriptors. Then, significant associations were determined between AUDPC, disease onset time, Gaussian parameters, maximum disease incidence and severity according to factor analysis. With these novel findings, we should be aware of descriptive value of wheat-stripe-rust progress variables. Such information will assist with stripe rust measurements for wheat breeding programs, yield loss estimation, development of disease control strategy, and epidemiological studies.

## 1 Introduction

Wheat stripe rust caused by *Puccinia striiformis* f. sp. *tritici* has been reported as a destructive disease worldwide. Under appropriate environmental conditions, severe epidemics of stripe rust can develop in particular on susceptible wheat crops. A noticeable number of environmental predictors of stripe rust epidemics across different geographical areas and wheat cropping systems have been identified previously. For instance, according to the large-scale study performed from 2009 to 2018 in Kermanshah province, Iran (Naseri and Sharifi 2020), the occurrence and intensity of stripe rust epidemics corresponded with the mean maximum relative humidity for Aban (from October 23 to November 21), mean minimum temperature for Esfand (from February 20 to March 20), number of icy days, number of rainy days, number of days with minimum temperatures within the range of 7–8 °C and relative humidity above 60%, and number of periods involving consecutive days with minimum temperature within the range of 6–9 °C and RH% > 60% during a 240-day period, from September 23 to May 21. At plot scale, Naseri and Marefat (2019) identified the disease-onset and maturity date, number of days with minimum temperatures within the range of 5–12 °C and RH above 60% during the growing season, resistance index, and planting date as the best agro-ecological indicators of wheat stripe rust development. Although comparative epidemiology of plant diseases has been known as a powerful tool to develop more effective disease management strategies (Jeger 2004), the characterization of wheat stripe rust progress curves has received little consideration.

**Figure 1:**
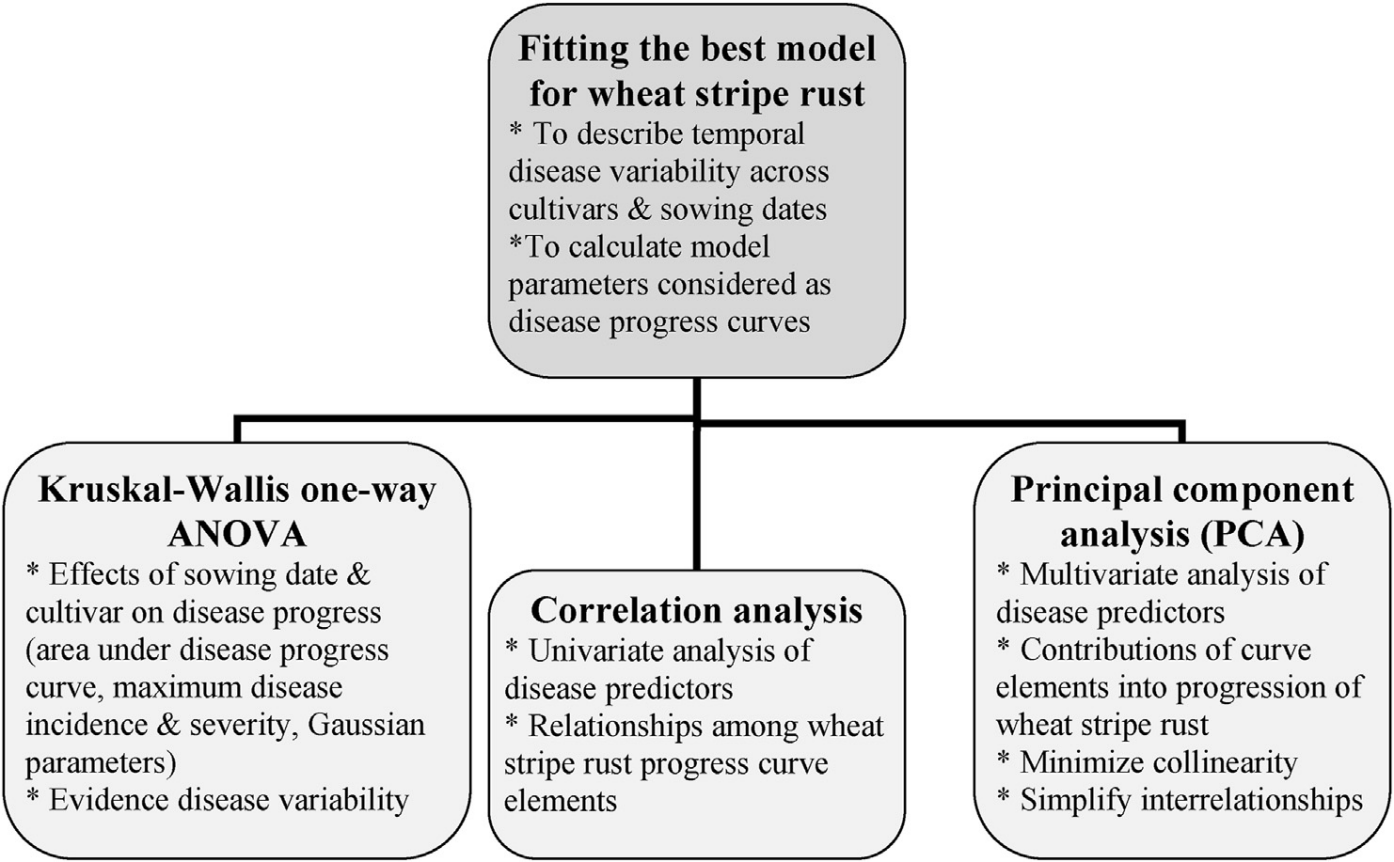
A flowchart summarizing epidemiological methods used to characterize progression of wheat stripe rust.

Most of previous attempts were made to compare the progression of stripe rust epidemics based on either apparent infection rate or the area under the disease progress curve (AUDPC) for evaluation of cultivar resistance and control method purposes (Kranz and Rotem 2012; Mengesha 2020). Kranz (1974) selected the best curve elements to characterize the progression of 40 different plant diseases studied over two seasons according to the PCA method. Pennypacker et al. (1980) also reported Weibull function as a flexible and simple model to study plant disease progression. However, further specific epidemiological studies are still required to fit stripe rust progress curves determined for a highly diverse range of disease levels over years and cultivars. Based on our preliminary examination of the parameters estimated by Weibull model, it was impossible to compare the development of wheat stripe rust between epidemic and non-epidemic (zero or low disease intensity) years on cultivars with various resistance levels. Hence, we have to consider only epidemic years and those cultivars with a notable susceptibility when using Weibull parameters to measure the rate and shape of stripe rust increase. In the comparison of the Gompertz and logistic functions to characterize 113 disease progress curves of nine pathosystems, Berger (1981) reported the superiority of the Gompertz model which avoided the curvilinearity generally associated with the logistic equation. Whereas, Hailu and Fininsa (2007) used a logistic model to estimate stripe rust progress rate in wheat cultivars treated with fungicides at different intervals in Ethiopia. Furthermore, Kranz (2003) attributed the strength of association between a curve element and the disease progress to the degree of heterogeneity within curve variables. This finding suggests that a better understanding of wheat rust dynamics depends on greater disease variability following the inclusion of influential agro-ecological parameters in epidemiological experiments (Naseri and Marefat 2019). Therefore, the identification of specific disease progress curve elements and the evaluation of relationships among disease growth-rate indicators for more accurate measurement of temporal dynamics of wheat stripe rust are still lacking. For this reason, a plot-scale attempt was made to examine: (1) which disease curve elements are associated with stripe rust epidemic variability across various wheat cultivars sown at different planting dates, (2) whether cultivar and planting date factors affected disease curve elements; (3) how strongly disease progress indicators are associated with each other, and (4) how much of disease variability is explained with the help of disease indicators under prevailing environmental conditions in Kermanshah province. To summarize the structure of this study, the best model was fitted to wheat stripe rust variability across different levels of cultivar resistance and planting date. In the next step, the effects of cultivar and planting date on the disease progress curve elements were examined during four growing seasons. Finally, interrelationships among the seven disease descriptors involving AUDPC, disease onset date, maximum disease incidence and severity, and parameters of the best model were explored (Figure 1).

## 2 Materials and methods

### 2.1 Epidemiological data collection

**Figure 2:**
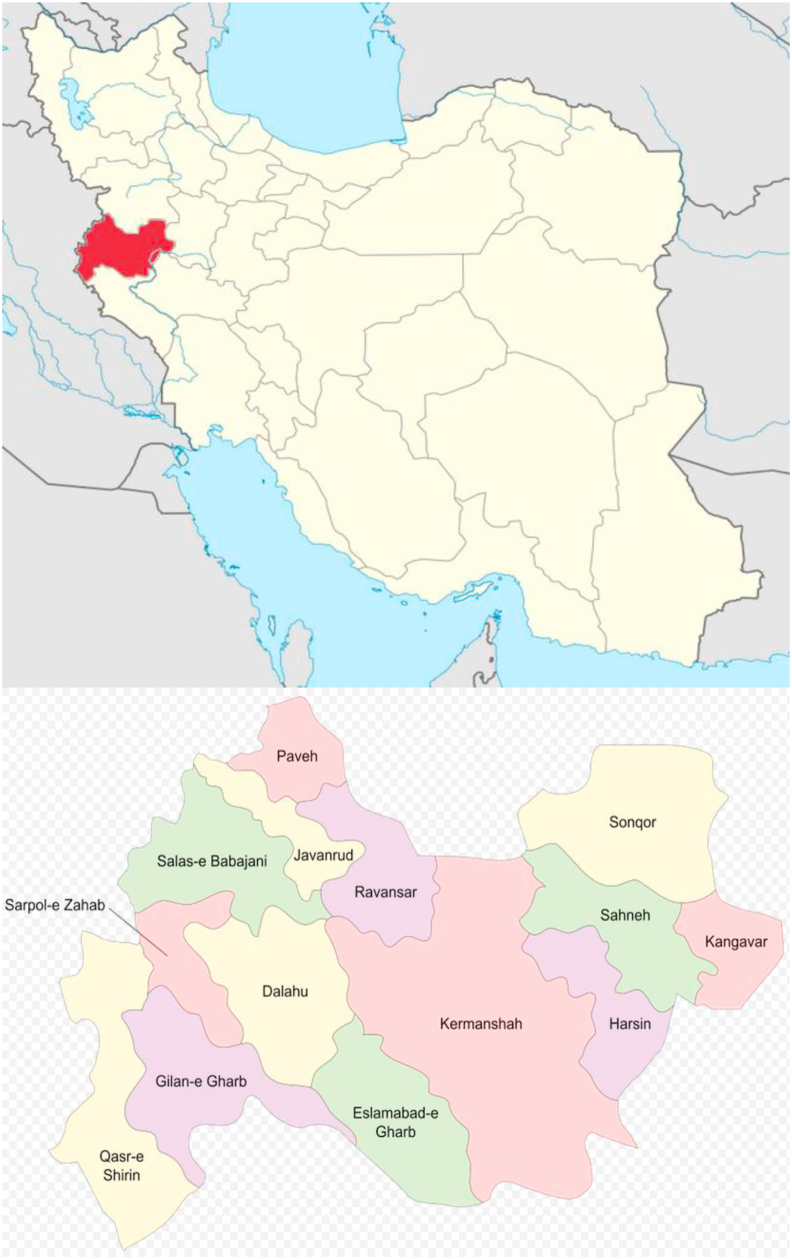
Maps of Iran (up), Kermanshah province and Eslamabad-e Gharb district (down).

From 2013 to 2017, stripe-rust-onset and disease development were examined in experimental winter wheat fields at Eslamabad-e Gharb Research Station (latitude 34˚7′ north, longitude 46˚28′ east; Figure 2) in four consecutive seasons (harvest years 2014, 2015, 2016, and 2017). The climatic characteristics for the Research Station of Eslamabad-e Gharb district, Kermanshah, were as follows: cool temperate climate, 479.8 mm annual rainfall, and 13.7 °C annual mean temperature. The experimental plots were not treated with fungicides even when stripe rust epidemics occurred. The plot size varied within the 6–24 m^2^ range depending on the study year. The four levels of planting dates (early autumn, mid-autumn, late autumn, and early winter) and eight wheat cultivars (Bahar, Baharan, Chamran II, Parsi, Pishgam, Pishtaz, Sirwan, and Sivand) were randomly applied to the experimental plots. According to the list of Iranian commercial cultivars registered every year for the study region, cv. Bahar was excluded from the research in the third and fourth years of field experiments. Cultivars were chosen as representative genotypes currently cultivated in main wheat growing areas of Iran. These bread wheat genotypes originated from the breeding program of the Seed and Plant Improvement Institute, Karaj, Iran, with the following characteristics: Bahar (pedigree ICW84-0008-013AP-300L-3AP-300L-0AP; susceptible to stripe rust), Baharan (pedigree KAUZ/PASTOR//PBW343; semi-resistant), Chamran II (pedigree Attila50Y//Attila/Bacanora; susceptible), Parsi (pedigree Dove”s”/Buc”s”//2∗Darab; semi-resistant), Pishgam (pedigree Bkt/90-Zhong87; resistant), Pishtaz (pedigree Alvand//Aldan/Ias58; resistant), Sirwan (pedigree PRL/2∗PASTOR; susceptible), and Sivand (pedigree KAUZ”s”/Azadiakd; susceptible). The above-mentioned experimental design resulted in a high within- and over-season heterogeneity in stripe rust development that was required to study disease progress curves. All agronomic practices (irrigation, fertilization, and control of weed and pest) were performed according to local standard recommendations.

The onset of stripe rust was determined when the first stripe rust pustule was evident on the leaf in each experimental plot. This disease indicator was classified to the early (before mid-spring) and late (after mid-spring) disease onset to improve its biological value for interpretation. Under prevailing environmental conditions in Eslamabad-e Gharb district of Kermanshah, only the early stripe rust spread before the middle of spring in coincidence with appropriate climate can result in severe epidemics in wheat crops (Naseri and Sharifi 2020). Disease incidence and severity were recorded at a 7–10 days interval as reported previously (Naseri and Marefat 2019). In brief, the incidence of stripe rust was measured as the percentage of diseased plants having long stripes of yellow pustules on the leaves for each of three random observations (100 plants per observation) per plot. The disease severity was determined as the percentage of leaf area postulating for the three youngest leaves (3–5 plants per plot). According to the highest disease severity rated over the four growing seasons from 2013-2017, the classification of stripe rust resistance level for the eight wheat cultivars was reconsidered as follows: semi-resistant for cv. Pishgam (<30%) and susceptible (>40%) for cvs. Bahar, Baharan, Chamran II, Parsi, Pishtaz, Sirwan, and Sivand.

### 2.2 Curve elements & statistical analysis

Stripe rust development in each experimental plot was characterized by eight variables associated with disease progress curves as follows: (1) disease onset as the time in days to initial yellow pustules on the leaf; (2) the AUDPC detected based on disease severity ratings using trapezoidal integration method by time in days (Madden and Nutter 1995); (3) maximum disease incidence observed over four-year disease assessments; (4) maximum disease severity; (5) *b*; (6) *m*; and (7) *s* Gaussian curve parameters which were considered as indicators of the rate of stripe rust increase according to the disease severity data. According to the regression analysis procedure, the fourteen standard curves were examined to model the progression of stripe rust on different wheat cultivars planted at different planting dates studied during four growing seasons as follows: exponential, a + br^x^; line plus exponential, a + br^x^ + cx; double exponential, a + br^x^ + cs^x^; critical exponential, a + (b + cx)∗r^x^; logistic, a + c/1 + exp(-bx + bm); generalized logistic, a + c/(1 + t∗exp(-bx + bm))^1/t^; Gompertz, a + c∗exp(-exp(-bx + bm)); linear-by-linear, a + b/(1 + d^x^); quadratic-by-linear, a + b/(1 + d^x^) + c^x^; quadratic-by-quadratic, a + (b + c^x^)/(1 + d^x^ + ex^2^); fourier, a + b∗sin(2π(x-e)/w); double fourier, a + b∗sin(2π(x-e)/w) + c∗(4π(x-f)/w); Gaussian, *a* + *b*∗Gauss((x – *m*)/*s*); double Gaussian, *a* + *b*∗Gauss((x – *m*)/*s*) + c∗Gauss((x – n)/*s*). All the statistical methods were performed using GENSTAT (Payne et al., 2009), which fits standard (nonlinear) curves by maximum likelihood. The co-efficient of determination (R^2^) and Fisher's (*F*) test were applied to testing goodness-of-fit (Brusco and Stahl 2005; Naseri and Marefat 2019). In the next step, predictions of stripe rust progression over time based on the above-mentioned standard curves were examined according to simple regression analysis of fitted and observed values (Berger 1981). Disease progress curves were characterized with the Gaussian model, which, compared with other models, gave the best fit to the disease progress data obtained from the four study years. A Gaussian model describes the bell-shaped curve used widely in statistical analyses to determine normal data distributions. As mentioned above, the Gaussian model can be written as:

Y = *a* + *b*∗Gauss((x – *m*)/*s*)

In this regression model, the term ‘*a*’ refers to the intercept (constant term); and the terms ‘*b*’, ‘*m*’ and ‘*s*’ indicate the three parameters of Gaussian model; and the term ‘x’ defines the time intervals (days) between consecutive disease assessments. To evaluate the effects of wheat cultivar and planting date on the descriptors of disease progress curves, the rankings of planting-date and cultivar factors were determined using a Kruskal-Wallis one-way ANOVA method. The significant differences in disease levels between rankings were examined according to the *H*-test results. This analysis compared the rankings for the AUDPC, maximum disease incidence and severity, the three Gaussian parameters according to planting date and cultivar factors. The two classes of early-optimum and late-very late were defined for the planting date factor.

A correlation analysis was performed to assess relationships between the continuous variables described as stripe rust progress curve elements. To ease the interpretation of stripe rust progression, a factor analysis based on correlation matrix was performed to determine the factor loadings for different disease variables (Madden and Pennypacker 1978). The loading values above 0.35 were used for statistical interpretation (Kranz 1974). An eigenvalue which indicates the proportion of total variance explained by each factor (Sharma 1996) is considered interpretable if it is greater than 1.0. Therefore, the four-year dataset on the progression of stripe rust was subjected to factor analysis to simplify the interpretation of interrelationships among the indicators of disease variability across wheat cultivars planted at different dates. Contributions of the disease indicators in principal factors according to the factors eigenvalues and variables loadings were considered as the importance of the disease component to describe the stripe-rust-wheat pathosystem.

## 3 Results

The progression of stripe rust on winter wheat grown at different planting dates was characterized in the four experiments performed in Kermanshah province. A series of standard curves was fitted to the disease severity data from each of the experiments to determine the model which best described the disease progression over time. The standard curve with the highest R^2^ and the lowest F values was considered the best fitted model for estimation of the temporal progress of wheat stripe rust. The Gaussian model, adopted for a four-season dataset from a highly diverse range of disease levels on wheat cultivars, with the rate parameters estimated for each experimental plot, evidenced to be the most satisfactory for stripe rust progression (Table 1). The test of a Gaussian model trend had significant results as follows: P *< 0*.001 for 2013–2014, 2015–2016, and 2016–2017, and P *= 0*.084 for 2014–2015 growing season. This result indicated that all of the stripe rust progress curves characterized for the disease severity data from the eight wheat cultivars planted at different planting dates over four growing seasons provided very good fits. Therefore, each stripe progress curve described the disease severity dynamics studied from the disease onset date to the physiological maturity of wheat plants. The percentage of variation explained (R^2^ value) by each Gaussian model developed for either of study years accounted for 0.97% in 2013–2014, 0.90% in 2014–2015, 0.96% in 2015–2016, and 0.94% in 2016–2017 wheat growing seasons. Estimation of the parameters of the Gaussian curves fitted to the disease severity data collected from the experimental plots were considered as descriptors of stripe rust progression on commercial wheat cultivars differing in resistance levels and planting dates. In the Gaussian model, which forms symmetric bell curves from a normally distributed data, the parameter *b* is the height of the curve's peak, the parameter *m* is the position of the center of the peak, *s* (the standard deviation) is the width of Gaussian bell. In addition to the three Gaussian parameters, the four variables of AUDPC, disease onset time, maximum disease incidence and severity were used as further disease progress curve elements. Mean, standard deviation, and range values were determined for the continuous variables considered to characterize the progression of wheat stripe rust at plot scale (Table 2).

**Table 1:** Standard models used to characterize stripe rust progress curves studied on wheat cultivars planted at different dates.

Models	2013–2014		2014–2015		2015–2016		2016–2017	
	*R*^2^	*F* prob.	*R*^2^	*F* prob.	*R*^2^	*F* prob.	*R*^2^	*F* prob.
Exponential	0.47	0.677	0.79	0.019	0.87	<0.001	0.47	0.630
Line + Exponential	0.73	0.136	0.87	0.213	0.92	0.003	0.82	0.017
Double exponential	0.77	0.907	0.88	nd^a^	0.93	nd	0.86	0.331
Critical exponential	0.67	0.443	0.87	0.193	0.93	0.002	0.86	0.001
Logistic	0.59	0.918	0.78	0.633	0.88	0.047	0.46	0.996
Generalized logistic	0.51	1.000	0.67	nd	0.88	nd	0.42	1.000
Gompertz	0.50	0.998	0.79	0.569	0.88	0.046	0.39	1.000
Linear-by-linear	0.46	0.718	0.79	0.022	0.87	<0.001	0.45	0.685
Linear-by-quadratic	0.78	0.011	0.88	0.175	0.93	<0.001	0.82	0.013
Quadratic-by-quadratic	0.95	<0.001	1.00	nd	0.92	nd	0.99	<0.001
Fourier	0.83	<0.001	0.88	0.155	0.94	<0.001	0.86	0.002
Double fourier	1.00	nd	0.20	0.112	nd	nd	0.99	nd
Gaussian	0.97	<0.001	0.90	0.084	0.96	<0.001	0.94	<0.001
Double Gaussian	0.99	nd	nd	nd	nd	nd	0.94	nd

a nd = Not detected by statistical procedure.

**Table 2:** Mean, standard deviation, and range values detected for continuous variables used to characterize stripe rust progression.

Variables	Mean	Standard deviation	Range
Area under disease progress curve	153.80	230.20	0.00 to 954.10
Maximum disease incidence	40.28	42.69	0.00 to 100.00
Maximum disease severity	20.57	26.36	0.00 to 100.00
Gaussian parameter *b*	215327.00	2059492.00	-23513.00 to 19969623.00
Gaussian parameter *m*	46.40	83.80	-10.80 to 285.00
Gaussian parameter *s*	4.22	10.25	0.00 to 57.00

The severe epidemic of wheat stripe rust developed in the experiment in 2015–2016 growing season. A commonly moderate (2013–2014 season) to mild (2014–2015 and 2016–2017 seasons) disease development was determined in the remainder of study years. According to the correlation analysis of disease curve elements (continuous variables), the AUDPC variable was significantly (P *≤ 0*.05) correlated with the maximum disease incidence and severity, and the Gaussian parameter *m* and *s* descriptors (Table 3). This result suggested that a greater AUDPC corresponded with a greater maximum disease severity and incidence, a higher center of the Gaussian-curve-peak (the parameter *m*), and a wider Gaussian bell (the parameter *s*). The maximum disease incidence indicator corresponded (P *≤ 0*.05) to the maximum disease severity, Gaussian parameters *m* and *s*. There were correlations (P *≤ 0*.05) between the maximum disease severity, Gaussian parameter *m* and *s*. Therefore, a greater maximum disease incidence and severity corresponded with a higher center of the Gaussian-curve-peak and a wider Gaussian bell. The variable of Gaussian parameter *m* was significantly related to the parameter *s*, suggesting the direct association of the height of the Gaussian-peak-center with the width of Gaussian bell. The correlation results were in agreement with the goodness-of-fit tests detecting the Gaussian function as the best model to describe the temporal progress of wheat stripe rust.

**Table 3:** Correlations between continuous descriptors of stripe rust progression in commercial wheat cultivars planted at various dates.

Variables	AUDPC	MDI	MDS	*b*	*m*	*s*
Area under disease progress curve (AUDPC)	1.00					
Maximum disease incidence (MDI)	**0.76**^a^	1.00				
Maximum disease severity (MDS)	**0.93**	**0.84**	1.00			
Gaussian parameter *b*	-0.01	0.15	0.12	1.00		
Gaussian parameter *m*	**0.71**	**0.53**	**0.63**	-0.03	1.00	
Gaussian parameter *s*	**0.67**	**0.51**	**0.65**	-0.00	**0.76**	1.00

a Bold numbers refer to significance at 0.05 probability level.

From the analysis of Kruskal-Wallis one-way ANOVA, the highest and lowest mean values for stripe-rust-AUDPC were detected in the early-optimum planting date of cvs. Sivand (mean = 70.21; rank = 1) and Pishgam (mean = 22.80; rank = 14), respectively (Table 4). According to the *H*-test results, mean AUDPC values for the both categories of planting date on cvs. Baharan, Chamran II and Sivand, and the early-optimum planting of cv. Sirwan were higher (mean adjusted *H* = 24.12; *P* = 0.030) than those for early-optimum plantings of cvs. Parsi and Pishgam. For the maximum disease incidence, the *H*-test (mean adjusted *H* = 29.94; *P* = 0.005) demonstrated a higher mean value in the early-optimum planting of cv. Sivand than those in the both planting-date categories of cvs. Parsi and Pishgam, and the early-optimum planting of cv. Pishtaz. For the maximum disease severity, the *H*-test (mean adjusted *H* = 28.22; *P* = 0.008) determined a higher stripe rust level in the early-optimum planting of cv. Sivand compared to the both planting-date categories of cvs. Pishgam and Pishtaz, and the early-optimum planting of cv. Parsi. The lowest and highest mean values for the maximum disease severity were observed in the early-optimum plantings of cv. Parsi (mean = 18.93; rank = 14) and Sivand (mean = 69.43; rank = 1), respectively. Furthermore, the early-optimum category of planting date for cv. Parsi reduced the AUDPC, maximum disease incidence and severity by nearly 50%. The early-optimum planting of cvs. Pishgam and Pishtaz decreased the AUDPC, maximum disease incidence and severity of stripe rust by 14–46%. The small differences in stripe rust AUDPC, incidence and severity between the two categories of planting date were determined for the four cultivars of Baharan, Chamran II, Sivand, and Sirwan. There was a lack of significant difference between the rankings of Gaussian parameters *b* (mean adjusted *H* = 17.24; *P* = 0.189), *m* (mean adjusted *H* = 11.61; *P* = 0.560), and *s* (mean adjusted *H* = 15.18; *P* = 0.290) detected for the cultivars and planting-date categories according to the *H*-test results (Table 4).

**Table 4:** Analysis of stripe rust variables examined by Kruskal-Wallis one-way ANOVA using *H*-test for commercial wheat cultivars planted at various dates.

Cultivars^a^	Planting date categories	Area under disease progress curve	Maximum disease incidence	Maximum disease severity	Gaussian parameters		
					*b*	*m*	*s*
Baharan	Early-Optimum	53.21 (7)^b^	54.93 (6)	51.43 (7)	49.86	58.50	52.64
	Late-Very late	56.00 (4)	56.71 (5)	58.36 (4)	69.93	60.71	66.43
Chamran II	Early-Optimum	57.64 (3)	59.00 (3)	57.43 (5)	45.21	48.57	41.14
	Late-Very late	54.71 (5)	57.86 (4)	59.86 (3)	58.86	53.00	54.43
Parsi	Early-Optimum	24.36 (13)	19.43 (13)	18.93 (14)	29.79	28.64	29.36
	Late-Very late	41.36 (9)	38.93 (10)	42.43 (9)	41.93	47.43	46.14
Pishgam	Early-Optimum	22.80 (14)	19.40 (14)	19.40 (13)	29.60	31.20	29.60
	Late-Very late	35.70 (11)	30.80 (12)	35.70 (11)	41.70	40.90	44.30
Pishtaz	Early-Optimum	34.43 (12)	35.14 (11)	34.50 (12)	37.29	35.29	36.71
	Late-Very late	39.93 (10)	45.71 (9)	40.79 (10)	45.64	43.93	44.93
Sirwan	Early-Optimum	53.36 (6)	52.00 (7)	54.43 (6)	50.29	48.79	44.43
	Late-Very late	45.21 (8)	46.86 (8)	47.50 (8)	44.36	52.21	47.50
Sivand	Early-Optimum	70.21 (1)	69.86 (1)	69.43 (1)	66.57	53.57	59.86
	Late-Very late	65.64 (2)	65.57 (2)	63.43 (2)	47.21	55.71	61.50
Mean adjusted *H*		24.12	29.94	28.22	17.24	11.61	15.18
Ranking *Chi P*		0.030	0.005	0.008	0.189	0.560	0.296

a Cultivar Bahar was excluded from this analysis because it was grown only in the first two years of study.

b Values inside parentheses refer to the significant ranking (*P* ≤ 0.05).

Based on the factor analysis, two principal factors accounting for 80% of the total variance characterized the progression of stripe rust developed on eight commercial wheat cultivars differing in planting date and disease resistance level studied from 2013 to 2017 in Kermanshah province (Table 5). The first principal factor accounted for 64.6% of the variance in stripe rust progress curves data. This factor showed the highest loading values (-0.43) for the negative contributions of the AUDPC and maximum disease severity descriptors. Based on this factor, only the disease onset time showed a positively moderate association. Furthermore, the maximum disease incidence, and Gaussian parameters *m* and *s* provided negatively moderate contributions in the first principal factor. These significant loading values (>0.35) evidenced the considerable linkages of the AUDPC, disease onset time, maximum disease incidence and severity, and Gaussian parameters *m* and *s* to the first principal factor. The second principal factor, which explained 15.8% of the data variance, evidenced the significant association of the Gaussian parameter *b* to estimate the progression of stripe rust during the wheat growing season. Moreover, in agreement with the correlation analysis, the loadings obtained for both of principal factors also demonstrated the significant associations of the AUDPC, disease onset time, maximum disease incidence and severity, and Gaussian parameters *b*, *m* and *s*. The first principal factor of PCA also demonstrated the reverse relationship of disease-onset time with the other negatively contributing stripe-rust variables, the AUDPC, maximum disease incidence and severity, and Gaussian parameters. This data suggested that an earlier stripe rust onset corresponded with greater AUDPC, maximum disease incidence and severity, a higher Gaussian peak center, and a wider Gaussian bell. According to the close contribution of disease onset time into the AUDPC, maximum disease severity, and Gaussian parameter *m* descriptors with the highest contributions into the first principal factor, the progression of stripe rust could be estimated based on the onset time (Table 5). Therefore, our factor analysis, correlation and *H*-test analyses evidenced the significant dependence of occurrence and development of stripe rust in wheat crops on the seven disease curve elements studied over a four-season research at plot scale.

**Table 5:** Factor analysis of stripe rust curve elements studied on commercial wheat cultivars planted at various dates.

Variables	Factors	
	1	2
Area under disease progress curve	**-0.43**	0.05
Disease onset time	**0.41**^a^	0.24
Maximum disease incidence	**-0.37**^a^	0.31
Maximum disease severity	**-0.43**^a^	0.22
Gaussian parameter *b*	-0.02	**0.85**^a^
Gaussian parameter *m*	**-0.42**^a^	-0.23
Gaussian parameter *s*	**-0.39**^a^	-0.13
Eigenvalues	4.52	1.11
Percentage variation justified	64.56	15.79

a A bold number indicates the significance of a loading ≥0.35 (Kranz, 1974).

## 4 Discussion

Although there has been much research reported on different predicting models for wheat stripe rust epidemics, the study of disease progress curves to characterize the disease progression over time deserves further attention. A quantitative description of stripe rust progress curves is needed to understand the structure of disease epidemics, comparative epidemiology, accurate disease monitoring on resistant cultivars, and yield loss estimation. Furthermore, the quantitative description of the plant disease progress in the field has been a subject of much interest in plant pathology and disease epidemiology from long ago to the present to improve the disease control via more effective cultural practices, more efficient fungicides, and more durable resistance genes (Kranz and Rotem 2012; Young 1978). To the best of our knowledge, there is no previous report of characterizing the structure of wheat stripe rust progression according to a combination of descriptors of AUDPC, disease onset time, maximum disease incidence and severity, and fitted curve parameters. Therefore, attempts were made in the current plot-scale research to evaluate the best curve elements for describing wheat stripe rust progression over a four-year period in western Iran, Kermanshah. To the best of our knowledge, this is the first record of the joint association of AUDPC, disease onset time, maximum disease incidence and severity, and three Gaussian parameters in the wheat-stripe-rust pathosystem.

Vanderplank (1963) recognized a number of important curve elements involving the disease onset time and the rate of disease progression. Kranz (1974) added more definition to the previous knowledge and identified 13 disease curve elements including AUDPC on a basis of factor analyses on curve elements of 40 diverse plant-pathogen pathosystems studied for two years. Young (1978) epidemiologically justified the assessment of general resistance in wheat cultivars commonly based on comparing the apparent infection rate (*r*) values in homogenously inoculated breeders' plots. Kranz (2003) also emphasized on the high final disease intensity as a useful curve element to be used for comparative epidemiology. Jeger and Viljanen-Rollinson (2001) recommended the AUDPC indicator for assessing quantitative disease resistance in crop cultivars, including the wheat-*Puccinia* pathosystem. In Ethiopia, Hailu and Fininsa (2007) considered the AUDPC and the logistic parameter to evaluate stripe rust progress in wheat cultivars treated with fungicides at different intervals. However, none of previous studies compared the importance of the above-mentioned disease progress curve elements for the stripe-rust-wheat pathosystem using the factor analysis. Hence, the present plot-scale findings evidenced the significant associations among the AUDPC, disease onset time, maximum disease incidence and severity, and three Gaussian parameters determined over a four-season study on wheat cultivars differing in resistance level and planting dates. This experimental design was applied to enhance variations in stripe rust progress trends as Kranz (2003) demonstrated that a greater variability in disease curves improves the strength of association between a curve element and the disease progress over time. Moreover, the current factor analysis and its first principal factor explained 80% and 64.6% of the variance in stripe rust progress curves data, respectively. This result suggested high predictive values of the AUDPC, disease onset time, maximum disease incidence and severity, and three Gaussian parameters to be used in future research on wheat stripe rust epidemiology and management.

The simple measurement of disease progress over the development of epidemics in the form of the proportion of diseased plants is often used to evaluate disease progress curves and determine disease increase patterns (Kranz 2003). Pennypacker et al. (1980) reported Weibull function as a flexible and simple model to study plant disease progression. Vanderplank (1963) introduced the term of apparent or absolute infection rate defined as a relevant *r* value to characterize the structure of plant disease epidemics. Then, Pennypacker et al. (1980) evidenced that *b* as the scale parameter of the Weibull probability density function is inversely correlated with the rate of disease increase. Campbell and Madden (1990) reported the goodness of fit in the estimation of progression for many plant disease epidemics. Likewise in southern Ethiopia, Mengesha (2020) fitted a logistic model to study the efficiency of four different fungicides to reduce stripe and stem rusts in five bread wheat cultivars at three experimental sites in 2018. In the comparison of the logistic (Vanderplank 1963) and Gompertz (Berger 1981) models for the goodness of fit, Mengesha (2020) evidenced a higher R^2^ for the logistic equation to describe temporal patterns of wheat stripe rust progress. Although this Ethiopean study involved the factors of cultivar, fungicide and location to increase the disease variability across field plots, considering only a one-year data appears inadequate for the temporal analysis of stripe rust progress in wheat crops. Therefore, attempts to fit standard curves for characterization of highly variable patterns of stripe rust increase in various commercial wheat cultivars planted at different times over a four-season research evidenced the goodness-of-fit for the Gaussian model in the current study. Moreover, the Gaussian parameters of *b*, *m* and *s* were considered as indicators of stripe rust increase for further statistical analyses to characterize the disease progress curves. This finding appears to be the first report of the Gaussian model fitted to stripe rust severity data collected from experimental wheat crops.

According to the present findings, the disease onset time indicated a noticeable association as significant as the AUDPC, the Gaussian parameters, and the maximum disease severity with the progression of stripe rust studied over four growing seasons of wheat at experimental plot scale. Such a remarkable contribution of the disease onset time in estimating wheat stripe rust progress may simplify future studies on predicting stripe rust outbreaks, screening for sufficiently resistant cultivars, measuring the durability of genotypic resistance, assessing fungicidal control efficiency, and estimating yield losses. Because the time of disease onset could be detected much earlier and easier, when compared to the multiple-point measurements of disease incidence and severity during the course of an epidemic and time-consuming calculations of fitted curve parameters. The present findings extended the current understanding of noticeable predictive value of the disease onset time to characterize stripe rust progress in wheat crops.

## 5 Conclusion

As conclusion, this is the first study to report the combined associations of the AUDPC, the disease onset time, the three parameters of Gaussian model, and the maximum disease incidence and severity to characterize the progression of stripe rust in wheat crops. Moreover, the current findings identified a simple-detected indicator for estimating the progression of wheat stripe rust according to the timing of disease onset. It should be noted that the applicability of these findings requires further experimentation and confirmation in other geographical regions with different agronomic practices, environments, and host plant and pathogen genotypes. Because the current research considered the two effective factors of wheat cultivar and planting date to increase stripe rust variability, the goodness-of-fit results for the Gaussian model are expected to be stable across different cropping systems. Furthermore, the remarkable associations of the disease spread and intensity descriptors with the Gaussian parameters demonstrated the high fitness of stripe rust progress model. From the disease management viewpoint, the joint analysis of cultivar and planting date factors evidenced that an earlier planting of three wheat cultivars could restrict the progression of stripe rust by 50%. Thus, the current novel findings may improve the efficiency of future predictions and disease progress assessments in other main wheat growing regions.

## Declarations

### Author contribution statement

Bita Naseri: Conceived and designed the experiments; Performed the experiments; Analyzed and interpreted the data; Contributed reagents, materials, analysis tools or data; Wrote the paper.

Homayoon Kazemi: Conceived and designed the experiments; Analyzed and interpreted the data; Contributed reagents, materials, analysis tools or data.

### Competing interest statement

The authors declare no conflict of interest.

### Additional information

No additional information is available for this paper.

## References

[bib1] Berger R.D. (1981). Comparison of the Gompertz and logistic equations to describe plant disease progress. Phytopathology.

[bib2] Brusco M.J., Stahl S. (2005). Branch-and-Bound Applications in Combinatorial Data Analysis. Statistics and Computing.

[bib3] Campbell C.L., Madden L.V. (1990). Temporal analysis of epidemics I. Description and comparison of disease progress curves. Introduction to Plant Disease Epidemiology.

[bib4] Hailu D., Fininsa C. (2007). Epidemics of stripe rust (*Puccinia striiformis*) on common wheat (*Triticum aestivum*) in the highlands of Bale, southeastern Ethiopia. Crop Protect..

[bib5] Jeger M.J. (2004). Analysis of disease progress as a basis for evaluating disease management practices. Annu. Rev. Phytopathol..

[bib6] Jeger M.J., Viljanen-Rollinson S.L.H. (2001). The use of the area under the disease-progress curve (AUDPC) to assess quantitative disease resistance in crop cultivars. Theor. Appl. Genet..

[bib7] Kranz J. (1974). Comparison of epidemics. Annu. Rev. Phytopathol..

[bib8] Kranz J. (2003). Comparison of temporal aspects of epidemics: the disease progress curves. Comparative Epidemiology of Plant Diseases.

[bib9] Kranz J., Rotem J. (2012). Experimental Techniques in Plant Disease Epidemiology.

[bib10] Madden L.V., Nutter F.W. (1995). Modeling crop loss at the field scale. J. Indian Dent. Assoc..

[bib11] Madden L.V., Pennypacker S.P. (1978). Analysis of epidemics using principal components. Proceedings of 3^rd^ International Congress of Plant Pathology, Germany.

[bib12] Mengesha G.G. (2020). Management of yellow rust (*Puccinia striiformis* f.sp. *tritici*) and stem rust (*Puccinia graminis* f.sp *tritici*) of bread wheat through host resistance and fungicide application in Southern Ethiopia. Cogent Food Agric..

[bib13] Naseri B., Marefat A. (2019). Wheat stripe rust epidemics in interaction with climate, genotype and planting date. Eur. J. Plant Pathol..

[bib14] Naseri B., Sharifi F. (2020). Predicting wheat stripe rust epidemics according to influential climatic variables. J. Plant Protect. Res..

[bib15] Payne R.W., Murray D.A., Harding S.A., Baird D.B., Soutar D.M. (2009). Genstat®.

[bib16] Pennypacker S.P., Knoble H.D., Antle C.E., Madden L.V. (1980). A flexible model for studying plant disease progression. Phytopathology.

[bib17] Sharma S. (1996). Applied Multivariate Techniques.

[bib18] Vanderplank J.E. (1963). Plant Diseases: Epidemics and Control.

[bib19] Young S.A. (1978). Quantitative epidemiology of stripe rust (*Puccinia striiformis* West.). Wheat Cultivars (*Triticum aestivum* Vill.) with General Resistance.

